# Antibody-mediated targeting of Claudins in cancer

**DOI:** 10.3389/fonc.2024.1320766

**Published:** 2024-02-02

**Authors:** Benjamin Vonniessen, Sébastien Tabariès, Peter M. Siegel

**Affiliations:** ^1^ Goodman Cancer Institute, McGill University, Montréal, QC, Canada; ^2^ Department of Medicine, McGill University, Montréal, QC, Canada; ^3^ Department of Biochemistry, McGill University, Montréal, QC, Canada; ^4^ Department of Anatomy & Cell Biology, McGill University, Montréal, QC, Canada; ^5^ Department of Oncology, McGill University, Montréal, QC, Canada

**Keywords:** Claudin, cancer progression, metastasis, antibody, antibody-drug conjugates, clinical trials

## Abstract

Tight junctions (TJs) are large intercellular adhesion complexes that maintain cell polarity in normal epithelia and endothelia. Claudins are critical components of TJs, forming homo- and heteromeric interaction between adjacent cells, which have emerged as key functional modulators of carcinogenesis and metastasis. Numerous epithelial-derived cancers display altered claudin expression patterns, and these aberrantly expressed claudins have been shown to regulate cancer cell proliferation/growth, metabolism, metastasis and cell stemness. Certain claudins can now be used as biomarkers to predict patient prognosis in a variety of solid cancers. Our understanding of the distinct roles played by claudins during the cancer progression has progressed significantly over the last decade and claudins are now being investigated as possible diagnostic markers and therapeutic targets. In this review, we will summarize recent progress in the use of antibody-based or related strategies for targeting claudins in cancer treatment. We first describe pre-clinical studies that have facilitated the development of neutralizing antibodies and antibody-drug-conjugates targeting Claudins (Claudins-1, -3, -4, -6 and 18.2). Next, we summarize clinical trials assessing the efficacy of antibodies targeting Claudin-6 or Claudin-18.2. Finally, emerging strategies for targeting Claudins, including Chimeric Antigen Receptor (CAR)-T cell therapy and Bi-specific T cell engagers (BiTEs), are also discussed.

## Introduction

**Figure 1:**
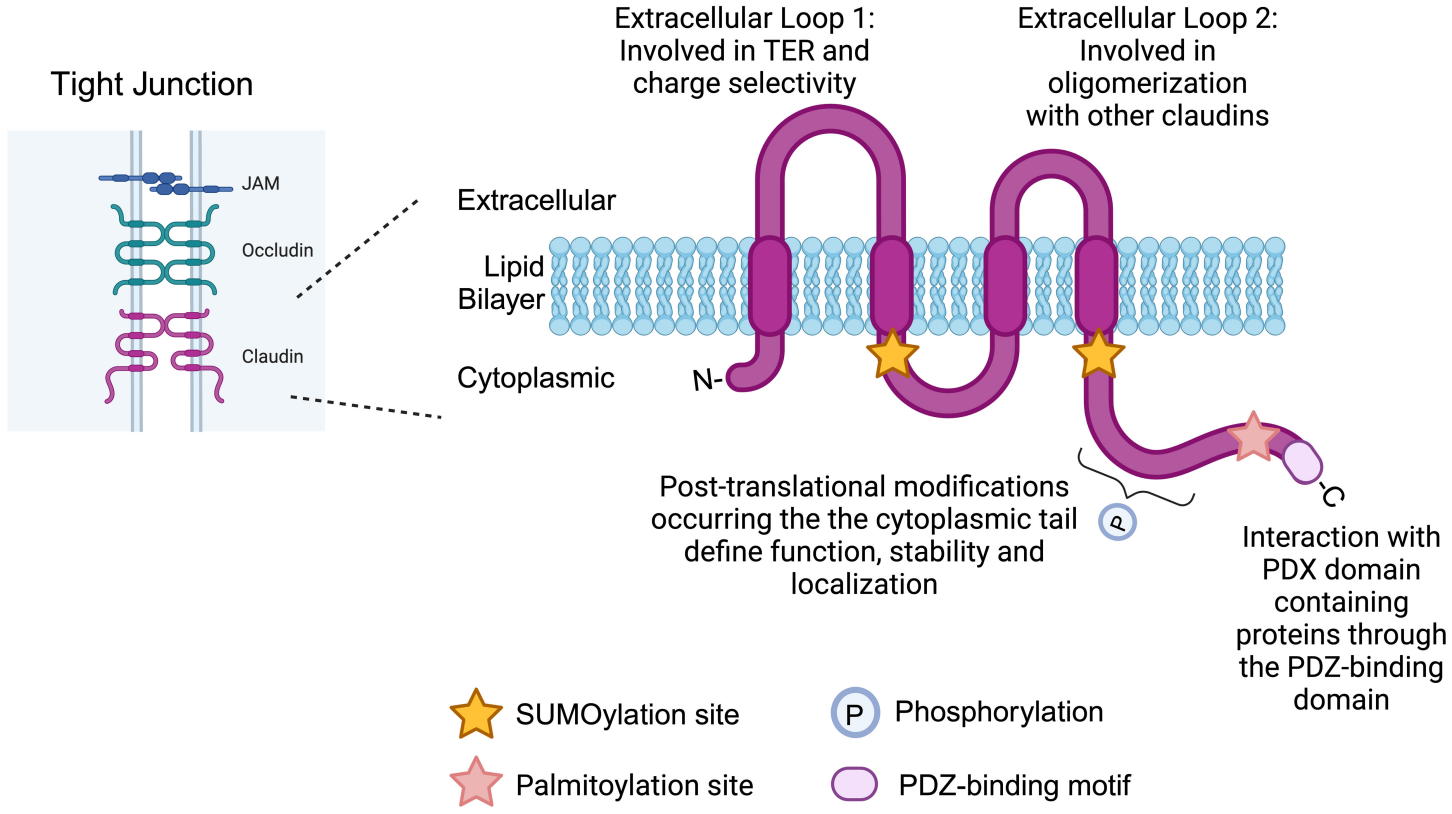
Structural organization of claudins. Schematic representation depicting the location of claudin proteins within apically positioned tight junctional complexes. The majority of claudin family members share a similar overall organizational structure that is composed of a short intracellular amino-terminal region, four transmembrane domains that form two extracellular loops and an intracellular carboxy-terminal cytoplasmic tail. The cytoplasmic tail of claudins contains several important sites for post-translational modifications, including phosphorylation, palmitoylation and SUMOylation. The extreme C terminus contains a PDZ-binding motif (YV) through which claudins bind to PDZ domain-containing proteins. Created with BioRender.com.

Claudins (CLDNs) are tetraspan transmembrane proteins that play key roles in the formation and maintenance of tight junctional complexes in epithelial and endothelial cells (1). Since the discovery of CLDN1 and CLDN2 in 1998 (2), the protein family has expanded to include 26 members in humans, ranging in size from 21 to 34 kDa (1, 3, 4). Claudins share a common structure comprised of the following: 1) a short N-terminal and longer C-terminal cytoplasmic region, 2) four transmembrane domains, 3) one intracellular loop and 4) two extracellular loops (ECL1, ECL2) (**Figure 1**) (5). Claudins engage in homo- or hetero-typic interactions with family members expressed on adjacent cells and are typically situated within apically located tight junctions that regulate paracellular permeability in addition to other barrier and fence functions (1).

Central to these functions are ECL1 and ECL2. Specifically, ECL1 mediates interactions between claudin family members that promotes 1) tightening of the paracellular cleft (CLDN1, CLDN3, CLDN4, CLDN5, CLDN8, CLDN11, CLDN14, CLDN19), 2) ion pore formation (CLDN2, CLDN7, CLDN10A/B, CLDN15, CLDN16) and 3) sealing/barrier functions that decrease paracellular ion permeability (CLDN4, CLDN5, CLDN8, CLDN11, CLDN14, CLDN19) (6). Pore ion specificity is determined by charged amino acids that, upon proper protein folding, face into the pore lumen (6). The functions of ECL2 are less well understood; however, ECL2 has been shown to self-associate, thereby narrowing the paracellular cleft. ECL2 is also the target of *Clostridium perfringens enterotoxin* (CPE) in a subset of claudin family members (CLDN3, CLDN4, CLDN6, CLDN7, CLDN8 and CLDN14) (6, 7).

Claudin isoform expression is dependent on tissue and developmental stage (8–10). Moreover, interactions between different claudin isoforms regulate junctional complex tightness (9). In this regard, certain tissues characterized by a high degree of permeability express elevated levels of pore forming claudins. Mouse kidneys have been shown to express CLDN1, CLDN2, CLDN3, CLDN4, CLDN7, CLDN8, CLDN10, CLDN11 and CLDN16 in segment-specific patterns, which determine local permeability (high or low) that underlie segment-specific filtration capacities in the kidney (11, 12). In contrast, tissues that require barrier impermeability, such as the duodenum facing the acidic chyme from the stomach, are enriched in tightening CLDN1, CLDN3, CLDN5 and CLDN8 (13). Expression of multiple claudin isoforms can also intrinsically regulate paracellular permeability through interference; CLDN4 has been shown to negatively regulate paracellular ion flow in the presence of CLDN2, CLDN7, CLDN15 and CLDN19 by disrupting their higher order structures (14).

Beyond their roles in modulating tight junctional permeability, claudins have been shown to play important roles in cancer progression. Claudins modulate cell survival, proliferation, metastatic progression and chemoresistance through interactions with PI3K/Akt/mTOR, Wnt and Erk signaling pathways, among others (15, 16). These functional properties can have seemingly opposing effects, resulting in claudins that act as a tumor suppressors or enhancers depending on 1) the tissue of origin, 2) the tumor stage and 3) the claudin of interest. These characteristics of claudins have been extensively reviewed elsewhere (5, 15, 17–21).

**Figure 2:**
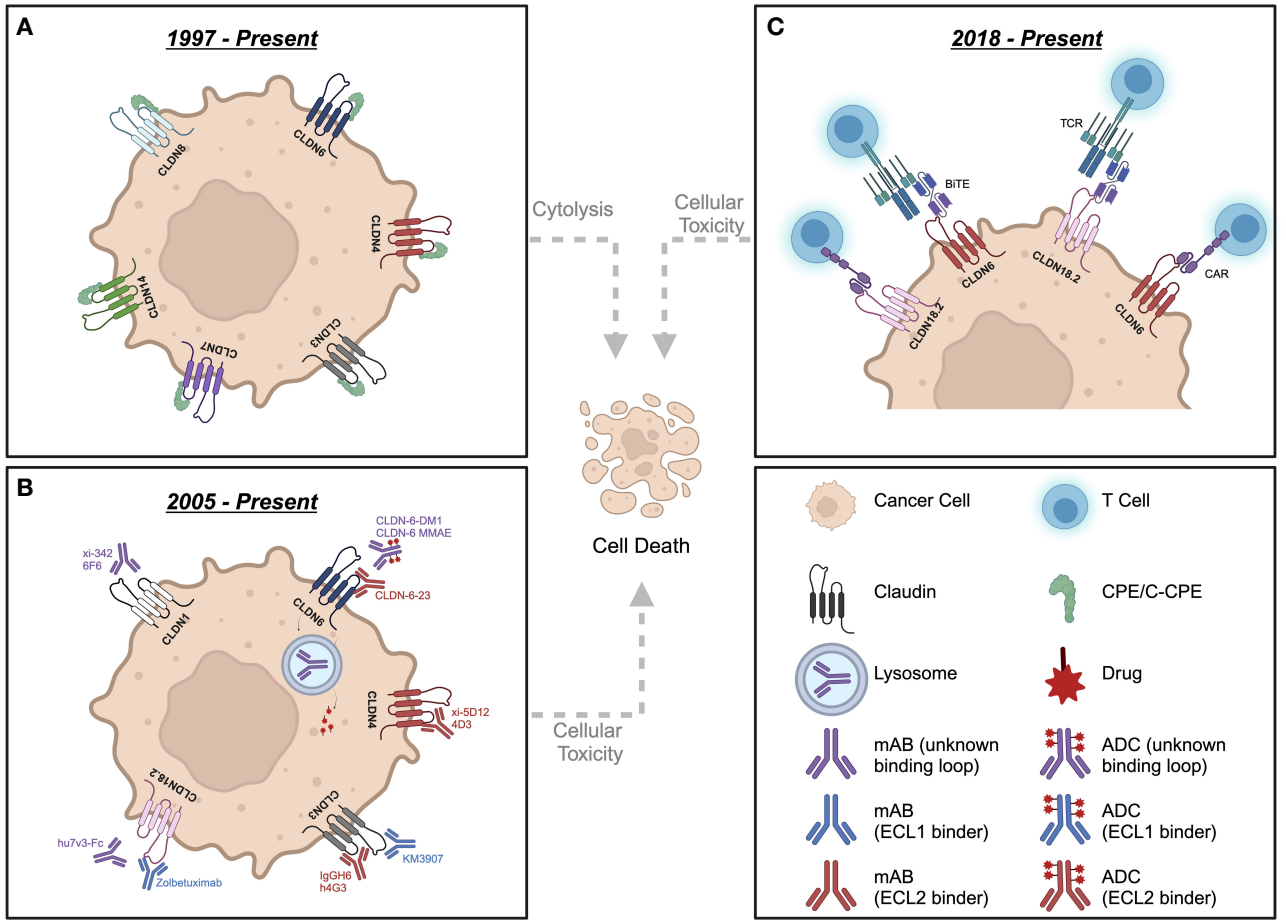
Schematic depicting various therapeutic strategies targeting claudin-expressing cancer cells. **(A)** Strategies involving CPE/C-CPE peptides. **(B)** Neutralizing mAbs and ADCs targeting distinct Claudin family members. **(C)** Strategies involving the development of CAR-T or BiTEs against CLDN6 and CLDN18.2. CPE, Clostridium Perfringens Enterotoxin; C-CPE, C-terminal fragment of Clostridium perfringens enterotoxin; mAb, monoclonal antibody; ADC, antibody-drug conjugate; CAR, chimeric antigen receptor; TCR, T cell receptor; BiTE, bi-specific T cell engager. Created with BioRender.com.

Overall, claudins represent a promising class of cancer therapeutic targets due to their extracellular accessibility, restricted tissue expression, unique localization patterns upon upregulation in malignancies and involvement in oncogenesis (22, 23). Different potential approaches through which Claudins may be targeted in cancer include the use of neutralizing antibodies to impair pro-tumorigenic and pro-metastatic functions of Claudins. These include interfering with claudin-mediated influences on cell signaling, cancer cell plasticity (epithelial to mesenchymal transitions) or homo- and heterotypic interaction between claudins aberrantly expressed by cancer cells or between cancer cell and stromal cells within the surrounding tumor microenvironment (5, 15, 17–21). Alternatively, ADCs can be used to exploit the increased expression of Claudins in a variety of cancer types to enhance the specific delivery of cytotoxic drugs to these tumors. Indeed, several claudin targeting strategies have been previously explored in the context of cancer (24–26). Initial attempts at targeting claudins involved the use of *Clostridium Perfringens* Enterotoxin (CPE), a bacterial product that utilizes its pore-and complex-forming domains to induce apoptosis in eukaryotic cells via Ca^2+^ influx (27–29). The last 30 amino acids within the C-terminal region of CPE have been identified as the region that binds CLDN3 and CLDN4, which is referred to as C-CPE (30). Thus, both the CPE and C-CPE reagents have been used to target claudins (**Figure 2A**) and have been reviewed extensively elsewhere (31–37). However, given that CPE is known to bind CLDN3 and CLDN4, along with CLDN6, CLDN7, CLDN8 and CLDN14 at lower affinities, a more specific therapeutic approach may be needed to target specific claudins in different disease indications (7).

The main goal of this review is to summarize current efforts in generating novel cancer therapeutics that target Claudins, which include 1) neutralizing antibodies, 2) antibody-drug conjugates (ADCs), 3) Chimeric Antigen Receptor (CAR)-T cell therapy or 4) Bi-specific T cell engagers (BiTEs). Claudin targeting antibodies are of increasing interest as the extra-junctional localization of claudins in tumor versus healthy tissue may afford a useful therapeutic window. Monoclonal antibody therapies can not only engage the immune system through antibody-dependent cellular cytotoxicity (ADCC) and/or complement dependent cytotoxicity (CDC)s, they can also be coupled to cytotoxic agents to generate ADCs against epitopes in both ECL1 and ECL2 (**Figure 2B**). Additionally, CAR-T or BiTE modalities directly stimulate an adaptive immune response at the tumor site (**Figure 2C**).

## Claudin-targeting antibodies: preclinical studies

The development of antibody-based therapeutics targeting Claudin proteins have relied heavily on pre-clinical animal models for two reasons. First, the functional importance of distinct Claudins in promoting tumor growth and progression to metastatic disease have been established using tractable animal models for various cancers. Second, the initial efficacy data for neutralizing antibodies or ADCs targeting Claudins have been generated using pre-clinical animal models. In this section, we summarize how such models enabled the development of therapeutic antibodies targeting CLDN-1, -3, -4, -6 or 18.2.

### Claudin-1

CLDN1 is dysregulated in multiple cancer types (19), including colorectal cancer (CRC), where it is overexpressed in primary tumors and CRC metastases (38). A humanized-mouse IgG1 antibody targeting CLDN1 (xi-342, **Table 1**) was found to significantly accumulate within HT-1080 xenograft tumors when compared to control IgG1 antibodies, which attenuated tumor growth through ADCC (39). Safety profiles for this antibody could not be evaluated given that xi-342 targets only human CLDN1; however, previous experiments using human liver chimeric mice treated with xi-342 revealed no significant toxicity by body weight and human albumin measurements (58).

**Table 1:** Preclinical studies characterizing Claudin-Targeting Antibodies.

CLDN	Ab clone	Target	Category	Antibody type	Tumor type	Reference
1	xi-342	Undetermined	Neutralizing	IgG1	Fibrosarcoma	(39)
	6F6	Undetermined	Neutralizing	IgG3k	Pancreatic, ovarian, hepatocellular and colorectal cancer	(38)
	6F6-MMAE	Undetermined	ADC	IgG3k	Colorectal cancer	(40)
	OM-7D3-B3	ECL1	Neutralizing	IgG2b, IgG4	Hepatocellular carcinoma	(41)
3	IgGH6	ECL2	Neutralizing	IgG1	Ovarian cancer	(42, 43)
	h4G3	ECL2	Neutralizing	IgG1	Breast, ovarian, colon, gastric, liver and pancreatic cancer	(44)
	KM3907, cKM3907	ECL1 (CLDN3/4)	Neutralizing	IgG2, IgG1 Fc	Ovarian cancer	(45)
4	KM3907, cKM3907	ECL1 (CLDN3/4)	Neutralizing	IgG2, IgG1 Fc	Ovarian cancer	(45)
	KM3934	ECL2	Neutralizing	IgG1	Ovarian and pancreatic cancer	(46)
	xi-5D12	ECL2	Neutralizing	IgG1	Colorectal and gastric cancer	(46, 47)
	4D3	ECL2	Neutralizing	IgG2b	Bladder, colorectal, gastric, pancreatic and breast cancer	(48–51)
6	CLDN6-2-DM1	Undetermined	ADC	IgG	Hepatocellular carcinoma	(52)
	CLDN6-MMAE	Undetermined	ADC	IgG2b	Germ cell tumors	(53)
	CLDN6-23-mAb	ECL2	Neutralizing	IgG1	Bladder, small cell lung and ovarian cancer	(54)
	CLDN6-23-ADC	ECL2	ADC	IgG1	Ovarian and bladder cancer	(54)
18.2	Zolbetuximab (IMAB362)	Undetermined	Neutralizing	IgG1	Pancreatic cancer	(55)
	hu7v3-FC	Undetermined	Neutralizing	IgG1	Gastric and pancreatic cancer	(56)
	CLDN18.2-307-mAb	Undetermined	Neutralizing	IgG1	Gastric and pancreatic cancer	(57)
	CLDN18.2-307-MMAE	Undetermined	ADC	IgG1	Gastric and pancreatic cancer	(57)

Similarly, an independent antibody generated against human CLDN1 (6F6, **Table 1**) was found to be highly specific, exhibiting no cross reactivity against murine CLDN1, human CLDN7 or CLDN8 (38). The neutralizing efficacy of 6F6 was assessed *in vitro* using colony formation assays involving multiple CLDN1 overexpressing cancer models (pancreatic: BXPC3, PANC-1; ovarian: SKOV-3, IGROV1; hepatocellular: HuH-7) as well as a xenograft model of CRC. *In vitro*, the number and size of colonies was reduced following incubation of all cancer cell models with 6F6. Moreover, 6F6 treatment significantly reduced CRC tumor growth and impairing metastasis to the liver *in vivo* (38). Researchers then linked 6F6 to monomethyl auristatin E (MMAE), an antimitotic agent (6F6-MMAE, **Table 1**). The ADC significantly decreased CRC growth when compared to the naked antibody in spheroid assays and resulted in a significant reduction in tumor growth compared to controls in a subcutaneous CRC model (40).

Using patient samples, it has been shown that chemotherapy resistance is significantly correlated with elevated CLDN1 expression and that *CLDN1* mRNA levels were upregulated in primary colorectal cancer tumors and metastases following chemotherapy (40, 59). As a result, the synergistic effects of the anti-CLDN1 ADC and oxaliplatin were assessed *in vitro* and *in vivo* in CRC models. The oxaliplatin dose was halved when combined with the ADC targeting CLDN1 (6F6-MMAE) and caused a significant reduction in tumor growth and prolonged survival when compared to oxaliplatin alone (40).

In hepatocellular carcinoma (HCC), CLDN1 is not only highly upregulated at both the mRNA and protein levels compared to matched healthy adjacent tissue, but it also localizes at extra junctional locations in cancer cells. Using an antibody raised against ECL1 of CLDN1 (OM-7D3-B3, **Table 1**) (41), researchers demonstrated specific binding to patient derived HCC cells compared to matched healthy tumor-adjacent tissue and showed efficacy against hepatoma cell lines in *in vitro* and *ex vivo* patient derived spheroid models including sorafenib- and nivolumab-resistant tumors (60).

### Claudin-3

CLDN3 is found upregulated in various cancer types, including ovarian, breast, colon, gastric, liver, and pancreatic cancer (61–66), and has been targeted in these contexts (42–45). A fully human IgG antibody (IgGH6, **Table 1**) was generated against CLDN3 (42, 43). Immunohistochemistry analysis revealed that IgGH6 bound to cell surface CLDN3 expressed on ovarian cancers cells, which was localized outside of cell-cell contact regions and readily internalized, undergoing a similar process as observed with C-CPE (42). Two additional human monoclonal antibodies, (h4G3 and KM3907, **Table 1**), have also been evaluated for their specificity and therapeutic efficacy in targeting CLDN3. The first, h4G3, was shown to specifically recognize the ECL2 of human and mouse CLDN3, with no evident cross-reactivity to other closely related human claudin family members. The second, KM3907, was specifically selected to target the ECL1 of both CLDN3 and CLDN4 but no other claudin family members. Both demonstrated ADCC *in vitro*, where h4G3 had dose dependent activity across multiple cancer cell lines that correlated with the levels of CLDN3 expression on target cells (44). Importantly, cKM3907 (fused to a IgG1 Fc domain) also had CDC activity and prevented measurable tumor formation of CLDN3 or CLDN4 transfected Chinese hamster ovary (CHO) when injected into SCID mice. Similar results were obtained using MCAS cells, a human ovarian cancer cell line that expresses both CLDN3 and CLDN4 (45).

### Claudin-4

CLDN4 has been found overexpressed in a variety of cancers (67). To date, targeting CLDN4 has been explored in pancreatic, ovarian, gastric, CRC, bladder or breast cancer mouse models (46–51, 68). Two humanized antibodies targeting the ECL2 of CLDN4 (KM3934 and xi-5D12, **Table 1**), demonstrated ADCC when co-cultured with human Peripheral Blood Mononuclear Cells (PBMCs) and ovarian cancer cells (KM3934) or human and mouse CLDN4 expressing cells (xi-5D12), respectively (46, 47). In xenograft models, KM3934 impaired the growth of ovarian (MCAS) and pancreatic tumors (CFPAC1) (46), while xi-5D12 significantly impaired tumor growth in mice bearing colorectal or gastric tumors (47). Interestingly, intravenous injection of xi-5D12 did not cause significant changes in weight loss nor to serum markers of liver and kidney damage compared to control cohorts (47).

Single agent treatment with a human monoclonal anti-CLDN4 antibody (4D3, **Table 1**), resulted in moderate effects on *in vitro* cell growth inhibition across bladder, CRC, gastric, pancreatic or breast cancer cells (48–51, 68). Increasing concentrations of anti-CLDN4 antibodies *in vitro* resulted in decreased invasion and significantly increased apoptosis, the latter effect was exacerbated when combined with chemotherapeutics (48, 49, 51, 68).

Interestingly, the intracellular accumulation of chemotherapy drugs was higher when T24 and RT4 bladder cancer cell lines were co-treated with 4D3 and chemotherapy compared to chemotherapy alone (48–50). Thus, *in vivo* experiments revealed synergistic effects on tumor growth inhibition and significant increases in survival when chemotherapy was combined with 4D3 (48–51, 68). Beyond the effects observed in primary tumors, the combination of 4D3 with chemotherapy in an experimental lung metastasis model of bladder cancer resulted in a significant reduction in lung metastasis when compared to treatment with 4D3 alone (49). Due to the enhanced antitumor effects achieved with 4D3 in combination with chemotherapeutics, researchers assessed the effects of combining 4D3 with half the dose of folfirinox (FFX) *in vivo*. Using a pancreatic ductal carcinoma mouse model, a similar degree of growth inhibition was observed with the combination of 4D3 plus a half-dose of FFX when compared to the full chemotherapy dose. Importantly, the reduced FFX dose, when combined with 4D3, did not cause the associated adverse effects observed in the full dose FFX cohort, and treated mice lacked symptoms of pancreatitis (48). These observations are reminiscent of the findings with anti-CLDN1 targeting antibodies that were combined with chemotherapy, which increased the amount of chemotherapy drug taken up by the cancer cells. Thus, an important use of anti-Claudin antibodies could be to limit chemotherapy-associated toxicities through dose reduction, while achieving a similar therapeutic effect.

### Claudin-6

CLDN6 has been investigated as a potential cancer therapy due to its specific enrichment in tumor tissue (69). In normal tissues, CLDN6 expression is restricted to embryonic cells during epithelial cell fate commitment and is otherwise transcriptionally silenced in adult tissues (54, 70, 71). As such, CLDN6 overexpression that is observed in ovarian, lung, endometrial, gastric, testicular and teratoma cancers makes this claudin a promising candidate for therapeutic intervention (72–78).

In addition, exogenous CLDN6 expression in HCC cell lines resulted in increased colony formation and cancer cell proliferation (52). Upregulation of CLDN6 expression is associated with sorafenib resistance, a standard therapy for advanced HCC, by slowing cell proliferation and increasing both YAP1/TAZ abundance and nuclear translocation (52). A CLDN6 targeting antibody has been linked to emtansine (CLDN6-2-DM1, **Table 1**), an anti-microtubular agent, with a drug-antibody ratio (DAR) of 3.6. CLDN6-2-DM1 impaired the growth of CLDN6 expressing HCC cell lines by suppressing YAP and TAZ levels and reducing the expression of liver progenitor markers. These *in vitro* results were validated using patient primary tumors and xenograft mouse models. As observed with CLDN1 and CLDN4 targeting antibodies, administration of CLDN6-2-DM1 combined with sorafenib treatment in a model of sorafenib resistant HCC caused significant growth inhibition of HCC tumors (52, 60).

Other cancers, such as germ cell tumors (GCT) exhibit similar patterns of signal pathway activation that are observed in HCC (53). As such, a CLDN6 antibody linked to MMAE (CLDN6-MMAE, **Table 1**) with a DAR of 3 was assayed in GCT cell lines expressing varying levels of CLDN6. CLDN6 was found highly expressed in a panel of germ cell cancer lines (10/17) (53). Treatment with the CLDN6-MMAE ADC induced similar levels of apoptosis, G2/M accumulation, and mitotic catastrophes when compared to unconjugated MMAE in cell line assays. The ADC also had greater cytotoxicity against a broad range of cell lines compared to the monoclonal alone. Importantly, when cultured with non-cancerous fibroblasts, CLDN6-MMAE resulted in less toxicity than unconjugated MMAE (53).

Another group generated a humanized mAb targeting ECL2 (CLDN6-23, **Table 1**) and showed significant inhibition of tumor growth in xenograft models of bladder cancer (UMUC4) and small cell lung cancer, with more modest responses observed in ovarian tumors (54). Body weight measurements revealed that the CLDN6-23 mAb was well tolerated (54). When conjugating this antibody to MMAE with a DAR of 4 (CLDN6-23-ADC, **Table 1**), no alteration in binding specificity, kinetics or internalization were observed compared to unconjugated antibody (54). CLDN6-23-ADC demonstrated dose-dependent *in vitro* growth inhibition in ARK2 and OVCA429 CLDN6-expressing cancer cells. Indeed, *in vitro* cell viability analyses performed on cancer cells post-treatment demonstrated that CLDN6-23-ADC was at least 10-fold more potent than CLDN6-23-mAb (54). *In vivo*, the ADC significantly reduced tumor cellularity by day 11 compared to control and mAb antibodies and elicited sustained anti-tumor responses with no measurable recurrence detected in up to 168 days post-treatment across UMUC4, ARK2 and OV90 xenograft models. These results were further validated in an ovarian PDX model, where the CLDN6-23-ADC displayed robust decreases in tumor volume, with all treated mice surviving for >100 days (54). Additionally, treatments were well tolerated, by body weight measurements, in all *in vivo* studies (54).

### Claudin-18.2

Expression of CLDN18.2, one of two isoforms of CLDN18, is restricted to differentiated cells of the gastric mucosa in healthy tissue; however, it is ectopically expressed by gastric, esophageal, pancreatic, lung and ovarian malignancies. Notably, CLDN18.2 positivity is frequently retained by metastatic lesions derived from gastric and pancreatic cancers (79–81). CLDN18.2 has also been shown to become upregulated following exposure of pancreatic cell lines to chemotherapy (55), a finding that has been observed with other claudins (40, 82, 83).

A neutralizing antibody targeting CLDN18.2 (Zolbetuximab/IMAB362, **Table 1**), induced ADCC and CDC mediated lysis of pancreatic cell lines engineered to overexpress CLDN18.2. *In vivo* pancreatic xenograft models revealed that Zolbetuximab significantly inhibited primary tumor growth and extended the survival of tumor bearing mice. Moreover, this anti-CLDN18.2 antibody significantly impaired the formation of lung metastases following tail vein injection of pancreatic cancer cells (55).

Using Zolbetuximab as a benchmark, another group generated humanized variable region heavy chain antibodies that, due to their smaller size, exhibit increased tissue penetration and tumor uptake compared to Zolbetuximab (56, 84). This agent (hu7v3-FC, **Table 1**) demonstrated higher ADCC efficiency compared to Zolbetuximab but similar CDC effects. hu7v3-FC was also shown to be effective in xenograft models of gastric and pancreatic cancer, resulting in significant tumor growth inhibition with no changes in body weight across all arms of the study (56).

Recently, technologies that use RNA-encoded antibodies have been employed to generate BNT141, which is composed of two pseudo-uridine modified mRNAs encapsulated within nanoparticles. The encapsulated RNAs are translated *in vivo* within the liver to produce an anti-Claudin-18.2 antibody (IMAB362/Zolbetuximab). BNT141 administration resulted in significant anti-tumor activity against a xenograft model of gastric cancer at lower i.v. dosages compared to IMAB362 (30μg vs 800μg respectively). Importantly, no overt clinical signs of gastric or systemic toxicity ware observed following BNT141 delivery. Pharmacokinetic studies revealed sustained expression of the antibody in the circulation of mice and nonhuman primates, which was dose dependent but not dose proportional. The analysis of serum harvested from primates receiving BNT141 demonstrated *in vitro* ADCC and CDC when combined with human PBMCs and CLDN18.2 expressing cells (85). This therapeutic agent is currently being investigated in a phase I/IIa clinical trial.

A new monoclonal IgG1 antibody, CLDN18.2-307-mAb, was recently described that is highly selective for human CLDN18.2. CLDN18.2-307-mAb exhibits >1,000-fold higher binding affinity when compared to zolbetuximab, using pancreatic cancer cells that endogenously express CLDN18.2 (HUPT4). *In vitro*, CLDN18.2-307-mAb possessed ADCC activity. In gastric and pancreatic cancer *x*enograft experiments using CD-1 nude mice, the antibody demonstrated superior growth inhibition compared to zolbetuximab. This effect was less striking in NSG mice that lack NK cells (57). This group then generated an ADC by linking MMAE via a cleavable linker to the CLDN18.2-307-mAb (DAR = 4). The fusion maintained the same binding efficiency observed with the mAb, and resulted in sustained and complete tumor regression using *in vivo* xenograft models of human pancreatic cancer (HUPT4 and PATU8998S) and a gastric model (SNU601), up to 7 weeks post treatment (57).

## Clinical trials assessing anti-Claudin antibodies

Despite a significant body of literature describing the development of antibodies targeting Claudin family members, at present only therapeutic agents targeting Claudin-6 and Claudin18.2 have progressed to efficacy assessment in clinical trials.

### CLDN6 (ASP1650/IMAB027)

Currently, one clinical trial has reported results regarding the efficacy of targeting CLDN6 in cancer (NCT03760081). ASP1650 (also known as IMAB027), an anti-CLDN6 mAb, was assessed in a phase II trial in the context of relapsed, treatment-refractory germ cell tumors (**Table 2A**). Nineteen patients were enrolled, the majority (63%) having received at least three prior lines of systemic therapy. Across the different dosages tested, none of the patients experienced a partial or complete response, with an overall response rate of 0 and the trial was terminated. Interestingly, 93.8% of patients were CLDN6 positive, as assessed by IHC staining of archival tumor tissues (86). Currently, one Phase I trial has been completed in ovarian cancer with results to be released (**Table 2B**) and there are two trials currently recruiting to explore targeting CLDN6 via ADC in CLDN6 positive advanced solid tumors (**Table 2C**).

**Table 2:** Clinical Trials Testing Cldn6 mAb/ADC.

**Table 2A:** Completed Clinical Trial with disclosed results.

Ab clone	ID	Category	Tumor type	Description	Study start date	Reference
ASP1650 (IMAB027)	NCT03760081	Neutralizing	Germ cell tumors	Phase II	2021	(86)

**Table 2B:** Ongoing Clinical Trial.

Ab clone	ID	Category	Tumor type	Description	Study start date
ASP1650 (IMAB027)	NCT02054351	Neutralizing	Advanced ovarian cancer	Phase I	2021

**Table 2C:** Recruiting Clinical Trials.

Ab clone	ID	Category	Tumor type	Description	Study start date
TORL-1-23	NCT05103683	ADC	Advanced solid tumors	Phase I	2021
DS-9606a	NCT05394675	ADC	Advanced solid tumors	Phase I	2023

### CLDN18.2 (Zolbetuximab)

The restricted expression of CLDN18.2 within gastric epithelium and its upregulation in a variety of solids cancers has fueled the rapid translation of anti-CLDN18.2 antibodies from pre-clinical studies into clinical trials (**Table 3**).

*Single agent trials:* Based on preclinical studies (55), Zolbetuximab was evaluated in a phase 1 clinical trial (NCT00909025, **Table 3**) to determine tolerated dosages and associated toxicities in patients with advanced gastroesophageal cancer. Fifteen patients were placed in five groups that received escalating doses of Zolbetuximab (33 to 1000mg/m^2^) in a single infusion. Primary objectives were to assess safety/tolerability for recommended phase II doses and secondary objectives were to assess the pharmacokinetic profile, immunogenicity and activity of Zolbetuximab. All dosages failed to cause major mucosal injury to gastric epithelia, with the most common reported adverse events (AE) being low grade nausea and vomiting (87).

A phase II trial, MONO (NCT01197885, **Table 3**) was conducted in 54 patients diagnosed with advanced gastroesophageal cancer using dosages in the range of 300-600 mg/m2. The primary objective was to evaluate overall response rate (ORR) at 11-12 weeks, with secondary objectives that included safety, tolerability, immunogenicity and pharmacokinetic profiles of the monotherapy. The MONO trial enrolled patients with recurrent or refractory locally advanced or metastatic CLDN18.2 positive gastric (GC), adenocarcinoma of the oesophagogastric junction (GEJ) or oesophageal adenocarcinoma. Patients received intravenous infusions of zolbetuximab for two weeks, with up to five infusion cycles. Out of the 43 patients that had assessable antitumor activity, clinical benefit rate [ORR + stable disease (SD)] was 23%. Specifically, four patients achieved a partial response and six achieved SD. Interestingly, ORR improved from 9% in the general patient population to 14% in patients that displayed moderate-to-high expression of CLDN18.2 (≥2+) in 70% of tumor cells (n=29) (89).

*Combination trials: FAST Trial (NCT01630083):* Given that chemotherapy remains the gold standard for treating advanced gastric cancer (94), another phase II trial of epirubicin, oxaliplatin and capecitabine (EOX) with or without zolbetuximab was conducted (90). A total of 246 patients were split into cohorts that received either EOX alone (arm 1, n=84), 600mg/m^2^ zolbetuximab + EOX (arm 2, n=77) or 1000mg/m^2^ zolbetuximab + EOX (arm 3, n=85). Benefits to progression-free survival (PFS) and overall survival (OS) correlated with CLDN18.2 positivity, where patients with tumors exhibiting moderate-to-high CLDN18.2 staining had the best outcome compared to EOX alone. PFS was 9 vs 5.7 and OS was 8.3 vs. 7.4 for combination therapy versus EOX alone respectively (90). Interestingly, the cohort receiving the high zolbetuximab dose exhibited a significant improvement in PFS compared to chemotherapy alone in patients with low-to-moderate CLDN18.2 expressing tumors, indicating that reduced levels of the target may be overcome by dose escalation. However, there was no benefit in OS and no significant improvement in OS or PFS in the moderate-to-high CLDN18.2 expressing patients (90). Similar to the MONO study (89), grade 1 and 2 adverse events (AEs) for nausea, vomiting, neutropenia and anemia were reported in both zolbetuximab arms, with haematological AEs reported across all treatment cohorts (90). In terms of patient reported outcomes, maintenance therapy with zolbetuximab lowered symptom burden and increased quality of life compared to EOX alone (95). Given that GC is very prevalent amongst Asian populations (96), a phase I trial (NCT03528629) of zolbetuximab in CLDN18.2-positive locally advanced/metastatic GC/GEJ patients from Japan was conducted, which yielded no new safety concerns (88).

*ILUSTRO Trial (NCT03505320)*: A phase II clinical trial was conducted to assess safety and efficacy of zolbetuximab alone (cohort 1, n=30), zolbetuximab in combination with mFOLFOX6 (cohort 2, n=21) or zolbetuximab plus pembrolizumab (cohort 3, n=3) in advanced GC/GEJ adenocarcinoma with moderate (≥50% but <75%) to high (≥75%) CLDN18.2+ staining. As expected, patients receiving zolbetuximab + mFOLFOX6 as a first-line therapy had an ORR of 71.4%; however, patients receiving zolbetuximab monotherapy or in combination with pembrolizumab as a third-line therapy had an ORR of 0%. Specifically, cohort 3 saw no patients achieving a complete nor partial response (91).

*SPOTLIGHT trial (NCT03504397):* Platinum fluoropyrimidine chemotherapy (folinic acid, 5-fluorouracil and oxaliplatin: FOLFOX) or capecitabine plus oxaliplatin (CAPOX) are standard chemotherapy regimens for HER-2 negative, locally advanced unresectable or metastatic GC/GEJ adenocarcinoma (97, 98). SPOTLIGHT was a global phase three trial conducted across 215 sites to evaluate the efficacy of Zolbetuximab plus chemotherapy (mFOLFOX6) versus chemotherapy alone in patients with CLDN18.2-positive, human epidermal growth factor receptor 2 (HER2)-negative, locally advanced unresectable or metastatic GC/GEJ. The primary endpoint was PFS, with secondary endpoints including: 1) OS, 2) time to confirmed deterioration, 3) duration of response (DOR), 4) safety and tolerability, 5) pharmacokinetics and 6) immunogenicity of zolbetuximab.

In a cohort of 565 globally distributed patients, Zolbetuximab + mFOLFOX6 significantly increased median PFS to 10.67 months versus 8.67 months for placebo + mFOLFOX6. At 24 months, PFS was 24% vs. 15% (92). OS was also significantly increased to 18.23 months in the combination arm versus 15.54 months in placebo + mFOLFOX6. At 24 months, OS was 39% vs. 28%, and at 36 months, OS was 21% vs. 9%. An objective response rate was observed in 48% of patients in both the zolbetuximab + mFOLFOX6 and placebo + mFOLFOX6 groups and the median duration of response was 8.51 months vs. 8.11 months, respectively. The placebo + mFOLFOX6 group reported grade 3 or worse AEs for nausea, vomiting and decreased appetite in 78% of patients in the placebo + mFOLFOX6 group and 87% in the zolbetuximab + mFOLFOX6 group (92).

*GLOW trial (NCT03653507)*: The efficacy of zolbetuximab was evaluated in a global phase III trial, GLOW, which used capecitabine and oxaliplatin (CAPOX) as a first line treatment for CLDN18.2-positive, HER2-negative, locally advanced unresectable GC/GEJ adenocarcinoma. The 507 patients selected for this trial possessed moderate-to-high CLDN18.2 IHC staining in ≥75% of the tumor. Participants were randomized to receive either zolbetuximab plus CAPOX or CAPOX alone. The primary endpoint for this study was PFS, with secondary endpoints including OS, ORR and DOR. In the intent-to-treat population, zolbetuximab plus CAPOX resulted in a statistically significant prolongment of PFS (14%) compared to the CAPOX alone arm (7%) at 24 months. Similarly, OS at 24-months was 29% in the zolbetuximab plus CAPOX group versus 17% in the CAPOX alone cohort. The study reported an ORR of 42.5% vs. 40.3%, with a DOR of 6.14 months vs. 6.08 months when comparing the zolbetuximab plus CAPOX cohort to the CAPOX alone cohort. In terms of safety, grade 3 AEs occurred in 72.8% of patients in the zolbetuximab plus CAPOX group versus 69.9% in CAPOX alone cohort. The most common AEs included vomiting, anemia, decreased neutrophil counts and nausea (93).

Importantly, there are currently several ongoing clinical trials that have yet to report results (**Table 3B**) and numerous trials that are actively recruiting to explore targeting CLDN18.2 as a single agent or as part of a combination therapy in CLDN18.2 positive advanced solid tumors (**Table 3C**).

**Table 3:** Clinical Trials Testing Cldn18.2 mAb/ADC.

**Table 3A:** Clinical Trials with disclosed results.

Ab clone	ID	Cancer type	Description	Study start date	Reference
Claudiximab (IMAB362)	NCT00909025	Advanced gastroesophageal cancer	Phase I	2009	(87)
Zolbetuximab	NCT03528629	Advanced gastroesophageal cancer	Phase I	2018	(88)
Claudiximab (IMAB362)	NCT01197885, MONO	Advanced gastroesophageal cancer	Phase II	2010	(89)
Zolbetuximab + EOX	NCT01630083, FAST	Advanced gastroesophageal cancer	Phase II	2012	(90)
Zolbetuximab + mFOLFOX6	NCT03505320, ILUSTRO	Advanced gastroesophageal cancer	Phase II	2018	(91)
Zolbetuximab + mFOLFOX6	NCT03504397, SPOTLIGHT	Advanced gastroesophageal cancer	Phase III	2018	(92)
Zolbetuximab + CAPOX	NCT03653507, GLOW	Advanced gastroesophageal cancer	Phase III	2018	(93)

**Table 3B:** Ongoing Clinical Trials.

Ab clone	ID	Cancer type	Description	Study start date
89Zr-NY005	NCT04989010	Solid tumors	N/A	2021
BNT141	NCT04683939	Advanced gastric, pancreatic, ovarian and biliary tract tumors	Phase I/IIa	2022
TST001	NCT05190575	Advanced biliary tract cancer	Phase II	2022

**Table 3C:** Recruiting Clinical Trials.

Ab clone	ID	Cancer type	Description	Study start date
CMG901	NCT04805307	GC/GEJ	Phase I	2020
124I-18B10(10L)	NCT04883970	Gastrointestinal tumors	N/A	2021
SYSA1801	NCT05009966	Advanced solid tumors	Phase I	2021
68Ga-ACN376	NCT05436093	Solid tumors	N/A	2022
68Ga-PMD22	NCT05937919	Advanced solid tumors	N/A	2023
IMC008	NCT05837299	Advanced solid tumors	Phase I	2023
SG1906	NCT05857332	Solid tumors	Phase Ia/b	2023

N/A, Not Applicable.

## Moving beyond antibody-based strategies: targeting CLDN6 and CLDN18.2

It is clear from the previous sections, that Claudin targeting strategies based on neutralizing antibodies or ADCs will continue to be an area of significant focus and investment. However, in recent years, additional strategies for targeting Claudins have emerged, which rely of the generation of Claudin-specific CAR-T cells or BiTEs.

### CAR-T cell therapy

CAR-T therapies are a form of adoptive T cell transfer immunotherapy in which T cells are engineered to express a chimeric cell surface receptor containing an antigen binding domain fused to signaling and costimulatory domains (in second and third generation CAR-Ts) that render them MHC-independent (99) (**Figure 2C**). Preclinical studies investigating the efficacy of CAR-T therapies for targeting CLDN6 and CLDN18.2 have demonstrated striking results (100, 101). Second generation CAR-Ts, containing costimulatory domains, raised against CLDN6 and CLDN18.2 have high specificity for their antigen with no cross reactivity to closely related claudin family members that can share up to 98% amino acid sequence homology (100, 101). Experiments have revealed that CLDN6 and CLDN18.2 CAR-Ts specifically recognize antigen expressing cancer cells, resulting in significant shrinking of tumors in xenograft models (100, 101). Interestingly, CAR-Ts have been detected in the circulation of “cured” mice up to 39 days post cell transfer (100), and CAR-Ts targeting murine CLDN18.2 showed no AEs, most likely due to the inaccessibility of CLDN18.2 localized in tight junctional complexes compared to the ectopic, extra junctional expression seen in tumor cells (101). The addition of an RNA vaccine containing mRNAs encoding CLDN6 (CARVac), when combined with human CLDN6 CAR-Ts, significantly enhanced CAR-T expansion, memory formation and efficacy compared to the delivery of CLDN6 CAR-Ts alone across multiple tumor models, including syngeneic gastric and lung models as well as a xenograft ovarian cancer model (100).

Other efforts have purified T cell receptor (TCR) genes from both CD4+ (DR4) and CD8^+^ (A2) T cells specific to CLDN6 that were obtained from a patient with ovarian cancer. CD4-transduced T cells and CD8-transduced T cells secreted multiple cytokines in the presence of B cells expressing their cognate antigen, and CD8-transduced T cells exhibited enhanced cytotoxicity against CLDN6 expressing cells compared to mock transduced T cells (102).

Most clinical trials investigating CAR-T therapies against CLDN6 or CLDN18.2 have yet to disclose results or are only just begun to recruit patients (**Tables 4**, **5**). However, interim results are available for a phase I trial investigating BNT211 +/- CARVac, a CLDN6 targeting CAR-T (NCT04503278, **Table 4B**) and a phase I trial conducted with CT041, a CLDN18.2 targeting CAR-T (NCT03874897, **Table 5**).

**Table 4:** Clinical Trials Testing Cldn6 CAR-T/BiTE.

**Table 4A:** Ongoing Clinical Trial.

Therapy	Ab clone	ID	Tumor type	Description	Study start date
BiTE	AMG 794	NCT05317078	Advanced solid tumors	Phase I	2023

**Table 4B:** Recruiting Clinical Trial.

Therapy	Ab clone	ID	Tumor type	Description	Study start date	Reference
CAR-TILs	N/A	NCT04842812	Advanced solid tumors	Phase I	2021	N/A
CAR-NK cell therapy	N/A	NCT05410717	Advanced solid tumors	Phase I, IIa	2022	N/A
CAR-T	N/A	NCT04503278	Advanced solid tumors	Phase I	2023	(103)
BiTE	SAIL66	NCT05735366	Advanced solid tumors	Phase I	2023	N/A
BiTE	BNT142	NCT05262530	Advanced solid tumors	Phase I, IIa	2023	N/A

N/A, Not applicable.

**Table 5:** Clinical Trials Testing Cldn18.2 CART/BiTE.

**Table 5A:** Completed Clinical Trial without disclosed results.

Therapy	Ab clone	ID	Cancer type	Description	Study start date
CAR-T	N/A	NCT04467853	Advanced solid tumors	Phase I	2020
BiTE	AMG 910	NCT04260191	GC/GEJ	Phase I	2020

**Table 5B:** Ongoing Clinical Trial.

Therapy	Ab clone	ID	Cancer type	Description	Study start date	Reference
CAR-T	N/A	NCT03159819	Advanced gastric and pancreatic adenocarcinoma	Phase I	2017	N/A
CAR-T	N/A	NCT03874897	Solid tumors	Phase I	2019	(104)

**Table 5C:** Recruiting Clinical Trial.

Therapy	Ab clone	ID	Cancer type	Description	Study start date
CAR-T	N/A	NCT04404595	Gastric, pancreatic or other digestive cancers	Phase Ib/II	2020
CAR-T CT041	N/A	NCT04581473	GC/GEJ and pancreatic cancer	Phase I/II	2020
CAR-TILs	N/A	NCT04842812	Advanced solid tumors	Phase I	2021
CAR-T	N/A	NCT05620732	Advanced solid tumors	Phase I	2022
CAR-T	N/A	NCT05583201	Solid tumors	Phase I	2022
CAR-T	N/A	NCT05472857	Advanced solid tumors	Phase I	2022
CAR-T	N/A	NCT05277987	GC/GEJ and pancreatic cancer	Phase I	2022
CAR-T	N/A	NCT05393986	Solid tumors	Phase I	2022
CAR-T	AZD6422	NCT05981235	Gastrointestinal tumors	Phase I	2023
CAR-T	N/A	NCT05952375	Advanced solid tumors	Phase I	2023
CAR-T	N/A	NCT05539430	Advanced GC/GEJ or pancreatic adenocarcinoma	Phase I	2023
CAR-T CT041	N/A	NCT05911217	Pancreatic cancer	Phase Ib	2023
TAC T-cells TACTIC-3	N/A	NCT05862324	Metastatic solid tumors	Phase I, II	2023

N/A, Not applicable.

Building from preclinical studies (100), researchers opened a clinical trial evaluating CLDN6 CAR-T +/- CARVac in patients with refractory metastatic CLDN6-positive solid tumors (germ cell and ovarian tumors predominantly), which has reported interim results for 22 of the patients enrolled (103). Patients were selected by CLDN6 expression, with a cutoff value of ≥50% of tumors cells displaying either intermediate or strong CLDN6 IHC staining. Patients received a single dose of CLDN6 CAR-T as a monotherapy (Dose Level 1 (DL1): 1x10^7^ cells; DL2: 1x10^8^ cells), or in combination with CARVac. Patients receiving autologous CLDN CAR-T infusion, comprising a mixture of CD4 and CD8 T cells, saw an ORR of 33%. ORR was positively correlated with peak expansion of CAR-T cells, as observed in the CT041 trial. The average time from baseline visit to infusion was 5.9 weeks, and upon receiving the infusion lymphodepleted patients at DL1 and DL2 saw maximal blood concentration of circulating CAR-Ts (Cmax) within 18 and 15.6 days respectively. Interestingly, in the DL2 cohort, the ORR was 46% with a clinical benefit of 85%. These results are striking given that the cohort of patients were refractory to a median of four previous lines of treatment. However, 86% of the patients experienced grade 3 AEs, most commonly neutropenia and leukopenia, and cytokine release syndrome occurred in 46% of patients (103).

At the interim report, patients receiving CT041 had completed more than 12 weeks of safety, efficacy and pharmacokinetic studies. Recruited patients had advanced stage GC/GEJ, pancreatic cancer and other primaries, with prior chemotherapy. Safety and efficacy were evaluated for 28 days following first infusion and followed up to a median of 8.5 months post apheresis (104). Measurable tumor regression was observed in 30 of the 37 patients enrolled on trial. In this study, 18 patients with GC that were non-responsive to previous lines of therapy displayed an overall response rate after receiving CAR-T infusion.

The overall response could be correlated with a Cmax. Interestingly, T cell subset frequencies detected in patients prior to infusion with CT041 greatly affected the measured Cmax values. For example, infusion of CT041 into patients with a lower proportion of terminally differentiated effector T cells was found to increase PFS. A higher proportion of central memory T cells present in patients before CT041 infusion increased Cmax values. Similarly, following first infusion, a higher Cmax was achieved when CT041 products contained lower frequencies of terminally differentiated effector T cells (104).

Additionally, CLDN18.2 expression was not downregulated following CT041 infusion, and 50% of patients previously unresponsive to anti-PD-1/PD-L1 displayed responses to CT041 (104). The most frequently reported AEs were preconditioning-related toxicities that resolved within a median of 4-9 days. Manageable off-target mucosal injury AEs were mostly of grade 1 or 2 and reported in only a subset (6/37) of patients (104).

CT041 was further investigated in two patients with metastatic pancreatic cancer. Both patients demonstrated a PR following CAR-T infusion; however, one (patient A) ultimately died due to disease progression. The other patient (patient B) saw a CR, with regression of their lung metastases and no progression of the primary tumor following 2 years of follow up. Interestingly, patient B maintained high levels of peripheral infused CAR-Ts until week 12, whereas CAR-T levels in patient A fell below detection by week 4 (105).

### BiTEs that target claudins

There is also growing interest in the generation of BiTEs, which are bi-specific antibodies containing single-chain variable domain fragments that simultaneously target CD3 and a tumor associated antigen (106) (**Figure 2C**), such as mouse CLDN6 (6PHU3) (107) or human CLDN18.2 (ZWB67) (108). Engagement of T cell receptors by 6PHU3 and ZWB67 results in T cell activation, proliferation and enhancement of cytotoxic effector phenotypes as revealed through co-culture experiments and gene expression profiling *in vitro*. Xenograft models in CD3 humanized mice treated with ZWB67 or NSG mice receiving PBMCs + 6PHU3 both showed significant regression of tumor and survival prolongation (107, 108). Interestingly, 6PHU3 also resulted in a 2- to 5-fold increase in immune cell infiltration across multiple subsets of CD3^+^ T cells compared to controls (107).

Other efforts have co-targeted CLDN18.2 and CD28 to overcome the lack of stimulation that may potentially limit BiTE-activated T cell responses. This BiTE significantly reduced tumor burden in a B16-OVA mouse melanoma model through activation of CD8^+^ T cells, as well as modulation of the tumor microenvironment by downregulating myeloid-derived suppressor cells and tumor-associated macrophages (109).

Comparisons between the efficacy of targeting CLDN18.2 using a BiTE versus an ADC have also been performed. The ADC was generated by conjugating auristatin to an anti-CLDN18.2 antibody with a DAR of 4. Both the anti-CLDN18.2 ADC and CLDN18.2 targeting BiTE, when administered individually, demonstrated *in vitro* cytotoxicity against BxPc3 and KATO III cell lines engineered to overexpress CLDN18.2. However, the ADC had an IC_50_ that was 2-10-fold lower than the BiTE, depending on the cancer cell line (80). In PDX xenograft models of pancreatic and gastric adenocarcinoma, a single dose of the CLDN18.2 ADC resulted in significant tumor growth inhibition. Treatment of the same gastric PDX model with the CLDN18.2 BiTE coupled with intra-peritoneal injection of 2 x 10^7^ expanded human T cells, caused a significant, dose-dependent reduction in tumor volume. Rat toxicity studies demonstrated that a 10mg/kg dose of the CLDN18.2 ADC was well-tolerated, with no clinical signs of toxicity observed. The BiTE, given at 0.34mg/kg, saw similar results (80). The clinical efficacy of BiTEs targeting claudins will become clearer as many of the ongoing or recruiting trials begin to report results (**Tables 4**, **5**).

## Emerging Claudin targets

Other claudin family members besides CLDN1, CLDN3, CLDN4, CLDN6 and CLDN18.2 have also been implicated in various stages of cancer progression, including metastasis. CLDN2 has been shown to be critical in mediating breast and colorectal cancer metastasis to the liver. *In vitro* selection of 4T1 cells to yield liver aggressive sub-populations revealed an enrichment of CLDN2 concomitant with loss of other tight junctional machinery components (110). Comparisons with matched primary tumors revealed an upregulation of CLDN2 expression in liver metastases and it was shown that CLDN2 is functionally required for attachment of cancer cells to hepatocytes through trans-homotypic interactions (111, 112). Early seeding and anchorage independent growth of breast cancer cells is mediated by CLDN2 interactions with downstream effector proteins, including recruitment of Afadin through the PDZ domain in CLDN2 (113). These interactions connect the junctional machinery to cell proliferation and survival pathways (114). CLDN2 is also an important promoter of CRC growth and spread to the liver and is specifically associated with poor prognosis replacement type liver metastases (115–117). In light of these observations, a IgG1 humanized anti-CLDN2 mAb (xi-1A2) was generated, which recognized human CLDN2, mouse CLDN2 and mouse CLDN3. This antibody promoted ADCC mediated cytotoxicity *in vitro* and attenuated tumor growth of HT-1080 xenografts *in vivo*. No overt adverse effects on body weight or increased serum markers of kidney dysfunction were observed (118, 119). Thus, the development of anti-CLDN2 neutralizing Abs or ADCs may represent a promising direction for therapies aimed at more effectively treating patients that develop liver metastases from various solid cancers.

As exemplified here by CLDN2, there are numerous claudins whose expression patterns or tumor/metastasis promoting functions make them potential clinical targets of interest including CLDN5 (120–122), CLDN7 (123–125), CLDN8 (126), CLDN9 (74, 127–129), CLDN10 (130–132), CLDN11 (133) or CLDN12 (134) (**Table 6**).

**Table 6:** Potential Claudin Targets.

CLDN	Tumor type	Reference
5	Pancreatic adenocarcinoma	(120)
	Serous ovarian adenocarcinoma	(121)
	Breast cancer	(122)
7	Pancreatic and colon cancer	(123)
	Colorectal cancer	(124)
	Nasopharyngeal cancer	(125)
8	Cervical carcinoma	(126)
9	Gastric adenocarcinomas	(74, 127)
	Hepatocellular carcinoma	(128)
	Endometrial cancer	(129)
10	Hepatocellular carcinoma	(130)
	Melanoma	(131)
	High-grade serous carcinoma	(132)
11	Head and neck cancer	(133)
12	Lung squamous cell carcinoma	(134)

## The potential of combination therapies incorporating anti-Claudin antibodies

The progress described in this review indicates that single agent therapies targeting Claudins can elicit therapeutic responses. While the SPOTLIGHT and GLOW trials have focused on the use of anti-Claudin neutralizing antibodies, the emerging utility of ADCs in oncology certainly bodes well for the clinical deployment of anti-claudin ADCs (135, 136). As technologies improve for delivery of neutralizing antibodies (e.g. vaccination strategies with mRNAs encapsulated in lipid nanoparticles) and designing ADCs (better linkers/payloads), it is likely that the efficacy of single agent anti-Claudin antibodies can be further improved. Importantly, data is already emerging that demonstrates significant improvements in anti-tumor responses when anti-Claudin antibodies are combined with chemotherapy. Further studies aimed at assessing the limits of chemotherapy dose reduction, when combined with anti-Claudin targeting antibodies, are warranted. Finally, the burgeoning field of immunotherapy will undoubtedly represent a potential area of synergy with anti-Claudin targeting agents. Preclinical studies has shown that treatment of patient derived HCC spheroids with CLDN1 mAb upregulated genes involved in immune effector function (60), and a BiTE targeting CLDN6 increased infiltration of CD3+ T cells in mice (107). Additionally, IHC analysis performed on patient samples have revealed correlations between CLDN18.2 expression, immune cell infiltration and PD-1 expression (137, 138). Thus, assessing synergies following the combination of anti-Claudin antibodies with immune checkpoint inhibitors may represent an area of intense interest moving forward.

## Conclusion

Taken together, there are characteristics that uniquely position claudins as exciting therapeutic targets in cancer. First, they provide extracellular targets that, in many cases, are restricted to specific tissues or defined developmental windows. Moreover, the extracellular loops of these proteins, which are bound by the many therapeutic modalities targeting claudins, are hidden within complex tight junctional structures that may reduce on target effects in normal cells. Second, claudins are often upregulated in the context of cancer, where they assume extra-junctional roles in cancer cells that have lost functional tight junctions, making them susceptible to therapeutic targeting.

Preclinical data has demonstrated that claudins assume roles beyond their primary function as tight junctional adhesion molecules in the context of cancer. Targeting claudins can effectively impair tumor proliferation and metastasis, disrupt major oncogenic intracellular signaling pathways and reduce chemotherapy resistance. Patient data has revealed that claudins are upregulated in a wide variety of cancer types. The use of *in vitro* and *in vivo* models has demonstrated that targeting CLDN1, CLDN3, CLDN4, CLDN6 and CLDN18.2 consistently reduces tumor burden across a multitude of different cancer types, with specific intra-tumoral accumulation and low rates of AEs. In addition, multiple studies have shown that claudin targeting acts synergistically to enhance the effect of chemotherapy regimens while minimizing associated toxicities.

Antibodies targeting claudins are beginning to show promise as cancer therapies; however, there remain several challenges that explain why only a limited number of claudin antibodies have advanced to clinical trials. Indeed, claudins are a large family of proteins with different claudin isoforms expressed by different tissues. Thus, achieving specificity for the claudin of interest, without affecting the function of other closely related claudins, can be challenging. As for any form of immunotherapy, antibodies may be associated with off-target toxicities, leading to adverse effects that limit therapeutic efficacy. Thus, ensuring the specificity of claudin antibodies and minimizing off-target effects is crucial for their safe and effective deployment in the clinic. Similarly, ensuring antibody delivery to the intended target site within the body remains challenging. This is particularly true for certain tissues or tumors that may possess barriers limiting antibody penetration (blood-brain barrier). Moreover, while antibodies may show promise in preclinical studies, translating their efficacy from animal models to humans can be complex. Differences in biology, pharmacokinetics and pharmacodynamics between species can impact the efficacy of the antibody observed in clinical trials.

However, these preclinical results have generated significant interest in targeting claudins as an effective therapeutic option. Large scale phase III clinical trials investigating antibody targeting of CLDN18.2 in gastric and gastroesophageal junction cancers have demonstrated significant improvements on progression free survival and overall survival in combination trials with standard of care chemotherapy regimens. This field has further expanded to include other strategies, such as ADC, CAR-T and BiTE approaches, which have all shown efficacy across diverse conditions and tumor types. CAR-T approaches that target CLDN18.2 or CLDN6 have recently entered into early phase I/II clinical trials and have also demonstrated efficacy in refractory patients with manageable safety profiles.

CLDN18.2 and CLDN6 have been the primary focus of current efforts to therapeutically target members of the Claudin family. The primary reason for selecting these particular family members stems from their highly restricted pattern of expression in normal tissues and aberrant expression in various tumors. Indeed, CLDN18.2 is an isoform that is specific to gastric epithelial cells; whereas, CLDN6 is a marker expressed in early embryonic stem cells that is silenced in differentiated tissues. These observations reveal a clear therapeutic window, which has accelerated the translation of various strategies to target these particular proteins into clinical trials. Moreover, these markers have not only been used as targets for drug delivery, but can also be exploited for diagnostic applications, such as imaging (139). Based on the promising results achieved thus far with CLDN6 and CLD18.2, there will be continued interest in exploring additional claudin family members that are emerging as important targets in different stages of tumor growth and metastatic progression.

## Author contributions

BV: Writing – original draft, Writing – review & editing. ST: Writing – review & editing. PS: Writing – review & editing.
